# Microstructural changes of the vestibulocochlear nerve in patients with Ménière's disease using diffusion tensor imaging

**DOI:** 10.3389/fneur.2022.915826

**Published:** 2022-09-26

**Authors:** Xiaojia Yuan, Xiaozhen Li, Yu Xu, Liqun Zhong, Zhanfeng Yan, Zhengguang Chen

**Affiliations:** ^1^Department of Chinese Medicine, Zhang Zhongjing College of Chinese Medicine, Nanyang Institute of Technology, Nan Yang, China; ^2^Department of Radiology, Dongzhimen Hospital, Beijing University of Chinese Medicine, Beijing, China; ^3^Department of Neurology, Dongzhimen Hospital, Beijing University of Chinese Medicine, Beijing, China; ^4^Department of Otolaryngology, Dongzhimen Hospital, Beijing University of Chinese Medicine, Beijing, China

**Keywords:** diffusion tensor imaging, Ménière's disease, vestibulocochlear nerve, microstructure, fractional anisotropy (FA)

## Abstract

**Objective:**

To evaluate the microstructural changes of the vestibulocochlear nerve in patients with Ménière's disease.

**Methods:**

A total of 26 subjects, 13 patients with MD and 13 healthy controls, underwent diffusion tensor imaging (DTI) on a 3T scanner. The independent sample *t*-test was used to compare the differences in fractional anisotropy (FA) and apparent diffusion coefficient (ADC) between the two groups. A Pearson correlation was used between DTI and the dizziness handicap inventory (DHI) scores.

**Results:**

There was a significant decrease in FA and an increase in ADC of the vestibulocochlear nerve in MD patients compared with healthy controls (*P* = 0.04, *P* = 0.001). FA had negative correlations with the DHI score (*r* = −0.62, *P* = 0.02) and DHI-functional score (*r* = −0.64, *P* = 0.02).

**Conclusion:**

These results are the first evidence of possible changes in the microstructure of the vestibulocochlear nerves in patients with MD. DTI is a potential technique for evaluating the vestibulocochlear nerve in patients with MD.

## Introduction

Ménière's disease (MD) is an inner ear disease and characterized by episodic attacks of vertigo, fluctuating hearing loss, tinnitus, aural pressure, and a progressive loss of audiovestibular functions (1). Endolymphatic hydrops (EH) was discovered and correlated with MD in 1938 (2). Recently, developments in gadolinium chelate (GdC)-enhanced MRI have provided a tool for visualizing endolymphatic hydrops non-invasively (3–6). With these new imaging techniques, EH can be used to confirm the diagnosis. However, there was no evidence that shown the correlation between EH and the severity and frequency of vertigo attacks (7–9). Currently, decreased hippocampal volume was found in Meniere's disease patients, and the volume was significantly correlated with severity of hearing and vestibular function of affected side (10). It indicates that MD may not only affect the inner ear. The syndromes of MD include vestibular function and hearing loss, and the vestibulocochlear nerve is a complex of cochlear and vestibular nerves. Thus, this research hypothesized there may be subtle changes in the vestibulocochlear nerve in MD patients and aimed to detect them with diffusion tensor imaging (DTI).

DTI can show the movement of neural fiber bundles under living conditions by describing and quantifying the extent and direction of water molecule diffusion (11, 12). In the healthy neural fiber bundles, the local water molecules that diffuse along the axon encounter smaller obstacles than those perpendicular to the axon (13, 14). Among the DTI parameters, the fractional anisotropy (FA) is a measure that essentially represents the SD of the eigenvalues, and the apparent diffusion coefficient (ADC) quantifies the amount of diffusion in all directions (15, 16). An FA is a scalar value between zero and one that describes the degree of anisotropy of a diffusion process. A value of zero means that diffusion is isotropic it is unrestricted (or equally restricted) in all directions. A value of one means that diffusion occurs only along one axis and is fully restricted along all other directions. FA is a measure often used in diffusion imaging where it is thought to reflect fiber density, axonal diameter, and myelination in white matter. ADC is a measure of the freedom of water molecular diffusion in the tissue environment. Research on cytotoxic edema suggests that the ADC is sensitive to a small change in the distribution of water between intra- and extra-cellular environments, and can be viewed as a probe of cell fluid electrolyte homeostasis. DTI has been used as a technique for various pathologies investigations, including stroke, amyotrophic lateral sclerosis, multiple sclerosis, and epilepsy in the central nerve system (17–20). Recent works evaluated the auditory pathway and facial nerves by DTI successfully, which indicated that DTI may be a potential tool for evaluating the integrity of the vestibulocochlear nerve by obtaining quantitative information (21–23). DTI parameters even can detect the development of white matter lesions before quantifiable changes in normal-appearing white matter (24). Moreover, it was reported that DTI parameters correlated strongly with neurophysiological measures in chronic inflammatory demyelinating polyneuropathy and lumbar spinal nerves (25, 26).

In this research, DTI was used to evaluate the vestibulocochlear nerve in MD, and the relation, between DTI parameters (FA and ADC) and clinical syndromes was also detected. The severity of vertigo was measured by the dizziness handicap inventory (DHI) scores. The outcomes of this research may contribute to the diagnosis of MD and understanding the pathogenesis of MD.

## Materials and methods

### Participants

With ethical approval and written informed consent, 13 patients with definite MD (7 female; mean ± SD age, 55.08 ± 12.72 years) and 13 healthy adults (7 female; mean ± SD age, 50.08 ±12.91 years) who were balanced in years and sexuality were recruited in our hospital for this study. The healthy control group were excluded if they had a history of vestibular symptoms or cochlear symptoms. Definite MD: Two or more spontaneous attacks of vertigo, each lasting 20 min to 12 h; audiometrically documented fluctuating low- to midfrequency sensorineural hearing loss (SNHL) in the affected ear on at least 1 occasion before, during, or after 1 of the episodes of vertigo; fluctuating aural symptoms (hearing loss, tinnitus, or fullness) in the affected ear; other causes excluded by other tests. All the patients were enrolled within 48 h of the vertigo attacks.

### MRI data acquisition

All MRI DTI data were acquired from all subjects on a 3T Siemens Verio clinical MRI scanner (Siemens Healthcare). T2^*^WI: thickness = 0.5 mm, TR = 1500 ms, TE = 289 ms, FA = 120°, FOV = 200 mm, matrix = 384 × 384. DTI sequence: slice thickness = 2.0 mm, FOV = 220 mm, matrix = 128 × 128, fractional anisotropy (FA) = 90°, b0 images = 65, diffusion gradient directions = 31, *b*-value = 1,000 s/mm^2^, TR = 10,500 ms, TE = 82 ms.

### Assessment of vestibular symptoms

The degrees of severity of dizziness were evaluated using the dizziness handicap inventory (DHI). The DHI is a 25-item questionnaire addressing the self-perceived handicapping effects of vestibular disease in terms of the patient's quality of life emotionally, physically, and functionally (27). Each score is evaluated by the patients separately. The DHI scores were 53 ± 20.76, DHI-functional were 23.69 ± 6.37, DHI-physical were 11.08 ± 8.07, and DHI-emotional were 18.15 ± 9.33.

### Data analysis

**Figure 1:**
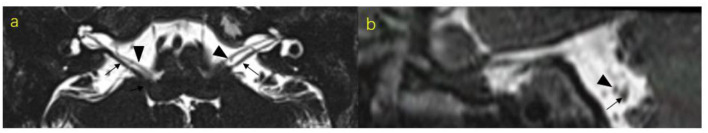
Vestibulocochlear nerve (arrows) and facial nerve (triangle) were separated on the T2-weighted axial **(a)** and sagittal **(b)** images from a representative subject.

**Figure 2:**
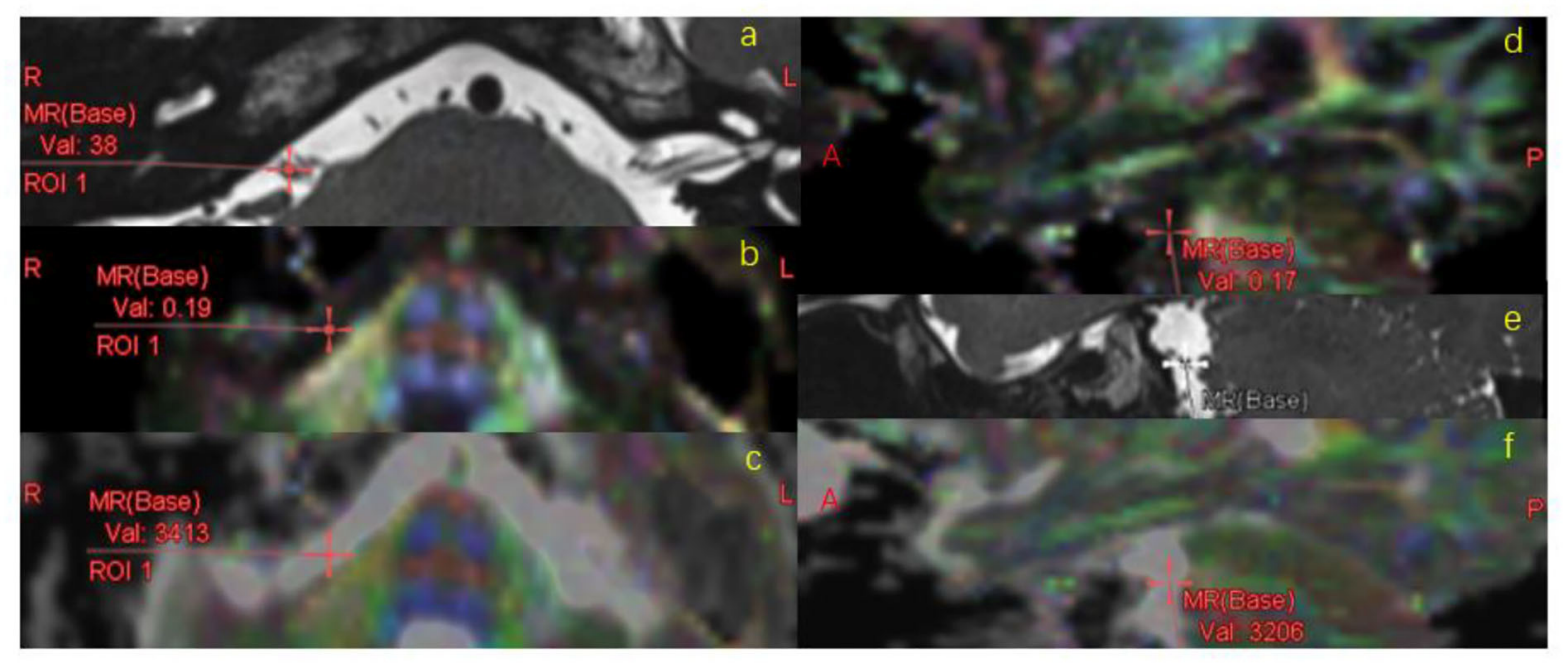
FA/ADC measurement. VCN was located on the T2 map **(a,e)**, registered to FA **(b,d)** map and ADC map **(c,f)** automatically, measured on FA/ADC map in the MR axial **(a–c)** and sagittal **(d–f)** images.

The diffusion tensor data was measured using a dedicated workstation (Ziostation2, Ziosoft Inc., https://ziosoftinc.com). FA and ADC maps were generated using the same software. This software provides a novel technique that can counterpoint the T2 map with the FA/ADC map by spatial location automatically. The 3D T2 image was rigidly registered to the DTI data automatically and midsagittal plane were transformed accordingly. FA and ADC values were obtained from the vestibulocochlear nerve (VCN). To determine whether the DTI parameters deviate from the normal range of values, the facial nerve (FN) was also measured. VCN and FN showed up clearly on the T2 image (Figure 1). They were located on the T2 map and measured on the FA map and ADC map by multi-points (an average of five points per nerve, Figure 2). The measurement was operated separately by two senior radiologists who were blinded between the control subjects and the MD patients. The average of each metric was taken forward into further analysis.

### Statistical analysis

Data were analyzed using SPSS 25 (IBM Corp). The FA and ADC values per nerve were compared using two sample *t*-tests to investigate the differences in patients and controls. The FA and ADC values from the side with hearing loss were compared separately against the other side to investigate whether there was a difference between the two sides in patients with unilateral deafness. A Pearson correlation coefficient analysis was performed on DTI parameters and DHI scores to examine the correlation between VCN changes and the degrees of dizziness. The receiver operating characteristic (ROC) curve was used to analyses the diagnostic efficacy of FA and ADC values. The statistical significance was set at *P* < 0.05. Data are presented as mean ± SD in the text.

## Results

### DTI of the interested nerves

The FA and ADC values of VCN and FN are shown in Tables 1, 2. In healthy controls, FA and ADC values of these nerves showed no significant difference in the left and right side.

**Table 1:** Clinical information and diffusion tensor imaging (DTI) parameters of MD patients.

**MD**	**1**	**2**	**3**	**4**	**5**	**6**	**7**	**8**	**9**	**10**	**11**	**12**	**13**
Sex	M	F	F	F	M	M	F	F	M	F	M	F	M
Age	51	51	68	58	32	65	56	54	27	66	59	64	65
Disease course	2 years	4 years	20 years	2 years	4 years	10 years	2 months	15 years	7 months	2 years	2 weeks	1 years	6 months
DHI	40	46	64	84	32	40	70	96	66	32	42	40	38
DHI-functional	18	20	24	32	16	24	32	36	26	16	20	24	20
DHI-emotional	12	20	30	36	16	4	16	32	20	12	14	10	14
DHI-physical	10	6	10	14	0	12	22	28	20	4	8	6	4
Stage of MD	I (R, L)	II (R)	III (R)	I (L)	I (R)	III (R)	I (L)	III (R, L)	I (L)	I (R, L)	I (R)	I (R, L)	I (R, L)
Frequency of attacks	Weeks	Months	Years	Months	Months	Years	Weeks	Years	Months	Years	Weeks	Months	Months
Number of attacks in last 3 months	10	2	1	3	2	1	6	1	2	1	2	2	1
FA of VCN (R)	0.19	0.14	0.15	0.14	0.33	0.20	0.13	0.17	0.26	0.29	0.24	0.16	0.28
FA of VCN (L)	0.21	0.20	0.15	0.13	0.25	0.24	0.16	0.13	0.26	0.22	0.31	0.25	0.14
ADC of VCN(R)	2.39	3.11	3.04	1.85	2.61	2.33	2.54	2.62	2.82	1.66	3.05	2.33	2.38
ADC of VCN (L)	2.56	3.00	2.65	2.80	2.88	3.04	1.97	2.57	2.91	2.90	1.81	1.90	3.15
FA of FN (R)	0.23	0.29	0.19	0.21	0.20	0.22	0.33	0.24	0.20	0.32	0.20	0.20	0.19
FA of FN (L)	0.24	0.19	0.23	0.32	0.24	0.24	0.45	0.18	0.43	0.37	0.30	0.18	0.18
ADC of FN(R)	2.42	2.89	2.83	2.92	3.03	2.42	1.43	3.51	3.60	2.79	3.17	2.49	2.88
ADC of FN (L)	2.45	2.51	2.56	2.25	3.42	3.33	1.97	3.84	2.97	3.07	2.69	2.35	2.90

FA, fractional anisotropy; ADC, apparent diffusion coefficient (10^−3^ mm^2^/s); VCN, vestibulocochlear nerve; FN, facial nerve; R, right side; L, left side.

**Table 2:** Diffusion tensor imaging (DTI) parameters of healthy controls.

**Healthy controls**	**Sex**	**Age**	**FA of VCN (R)**	**FA of VCN (L)**	**ADC of VCN (R)**	**ADC of VCN (L)**	**FA of FN (R)**	**FA of FN (L)**	**ADC of FN (R)**	**ADC of FN (L)**
1	F	43	0.27	0.25	2.22	2.30	0.18	0.33	2.91	1.82
2	M	32	0.25	0.18	1.67	1.18	0.23	0.28	2.65	2.32
3	M	41	0.24	0.28	2.26	2.32	0.18	0.18	2.45	2.35
4	F	41	0.24	0.26	2.46	2.49	0.19	0.26	2.85	1.86
5	F	46	0.24	0.20	2.79	3.29	0.27	0.19	2.13	2.64
6	M	68	0.18	0.24	2.95	2.67	0.19	0.16	2.88	3.10
7	M	48	0.21	0.35	2.23	2.21	0.18	0.31	2.53	2.55
8	F	67	0.25	0.19	1.06	2.91	0.35	0.18	2.31	2.90
9	F	60	0.26	0.28	2.54	1.81	0.27	0.16	2.10	3.18
10	M	58	0.34	0.26	1.76	1.03	0.27	0.36	2.62	1.74
11	M	61	0.24	0.23	1.92	1.30	0.21	0.30	1.14	1.78
12	F	28	0.37	0.25	1.80	2.30	0.39	0.27	2.02	1.55
13	F	58	0.30	0.18	1.00	1.18	0.41	0.27	2.45	2.16

FA, fractional anisotropy; ADC, apparent diffusion coefficient (10^−3^ mm^2^/s); VCN, vestibulocochlear nerve; FN, facial nerve; R, right side; L, left side.

### Comparison of DTI parameters

**Figure 3:**
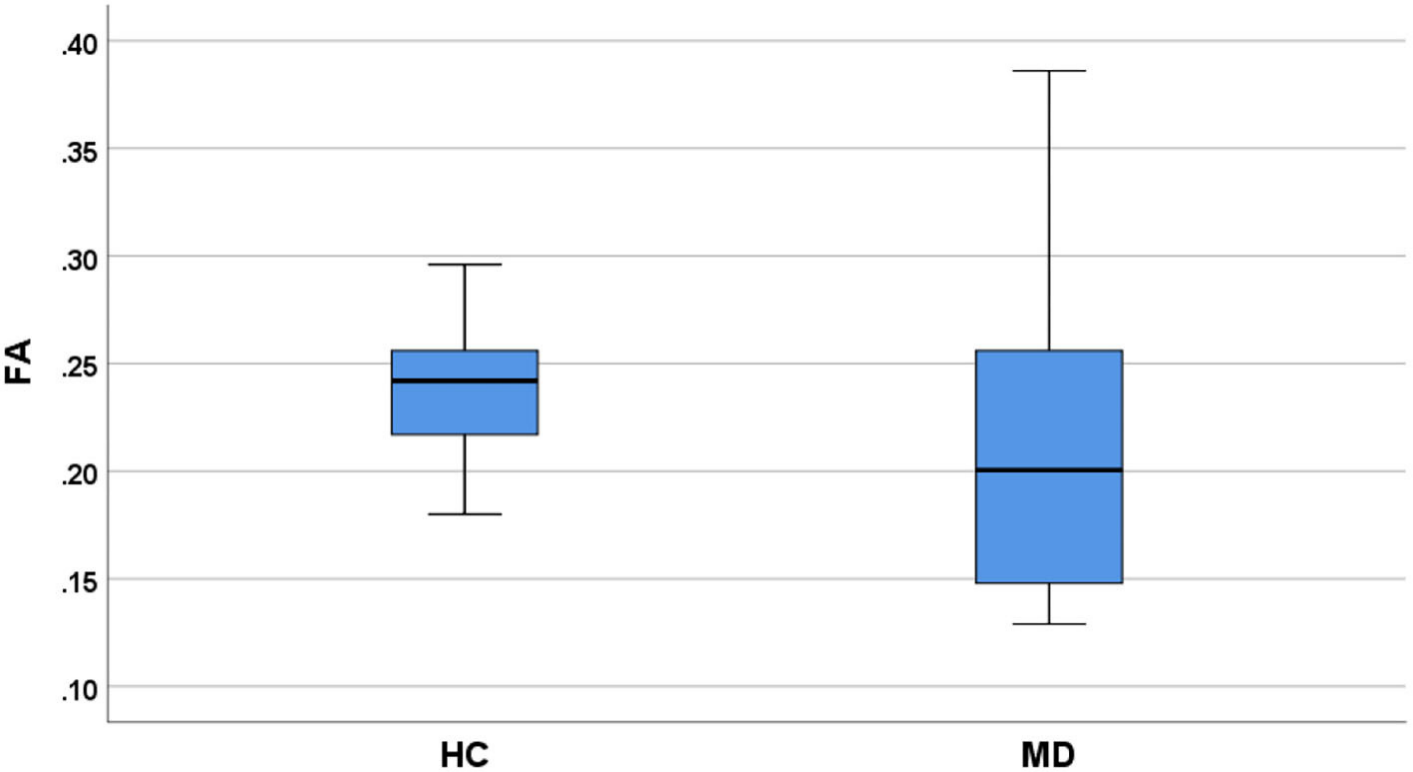
Comparison of FA values (VCN) between MD patients and healthy controls (HC).

**Figure 4:**
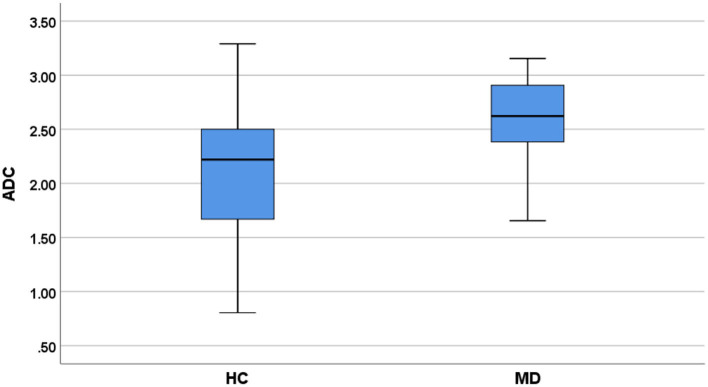
Comparison of ADC values (VCN) between MD patients and healthy controls (HC).

**Figure 5:**
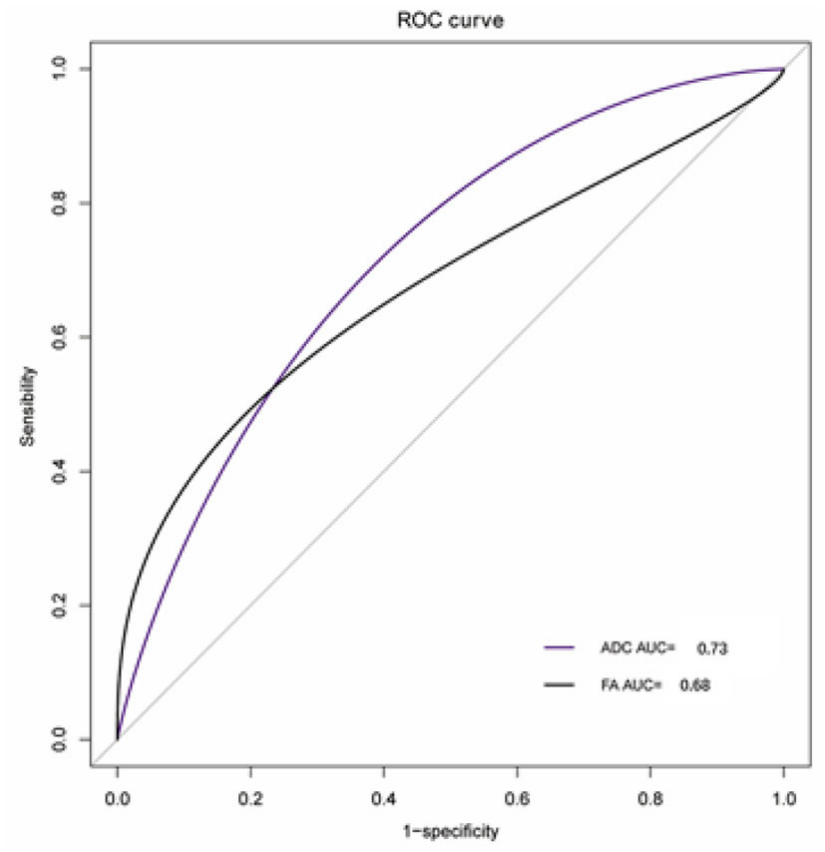
The ROC curves of FA and ADC values.

FA of VCN was lower in patients (0.21 ± 0.01) compared with controls (0.24 ± 0.01, *P* = 0.04, Figure 3). ADC of VCN was higher in patients (2.59 ± 0.09) compared with controls (2.06 ± 0.13, *P* = 0.001, Figure 4). No differences were detected in FA and ADC values from FN, CN, and VN in the patients and healthy controls. Further, the FA of VCN was significantly lower than that of the facial nerve (0.24 ± 0.08, *P* = 0.02). Eight patients showed unilateral hearing loss, FA and ADC of the vestibulocochlear nerve showed no significant difference between the deafness side and the other side (*P* = 0.73, *P* = 0.56). Based on ROC curve analysis, the FA cutoff point was 0.18, with the area under the ROC curve (AUC) 0.68, sensitivity 0.42, specificity 1; the ADC cutoff point was 2.56 × 10^−3^ mm^2^/s, with AUC 0.73, sensitivity 0.62, specificity 0.81 (Figure 5).

### DTI parameters correlate with assessment of vestibular symptoms

**Figure 6:**
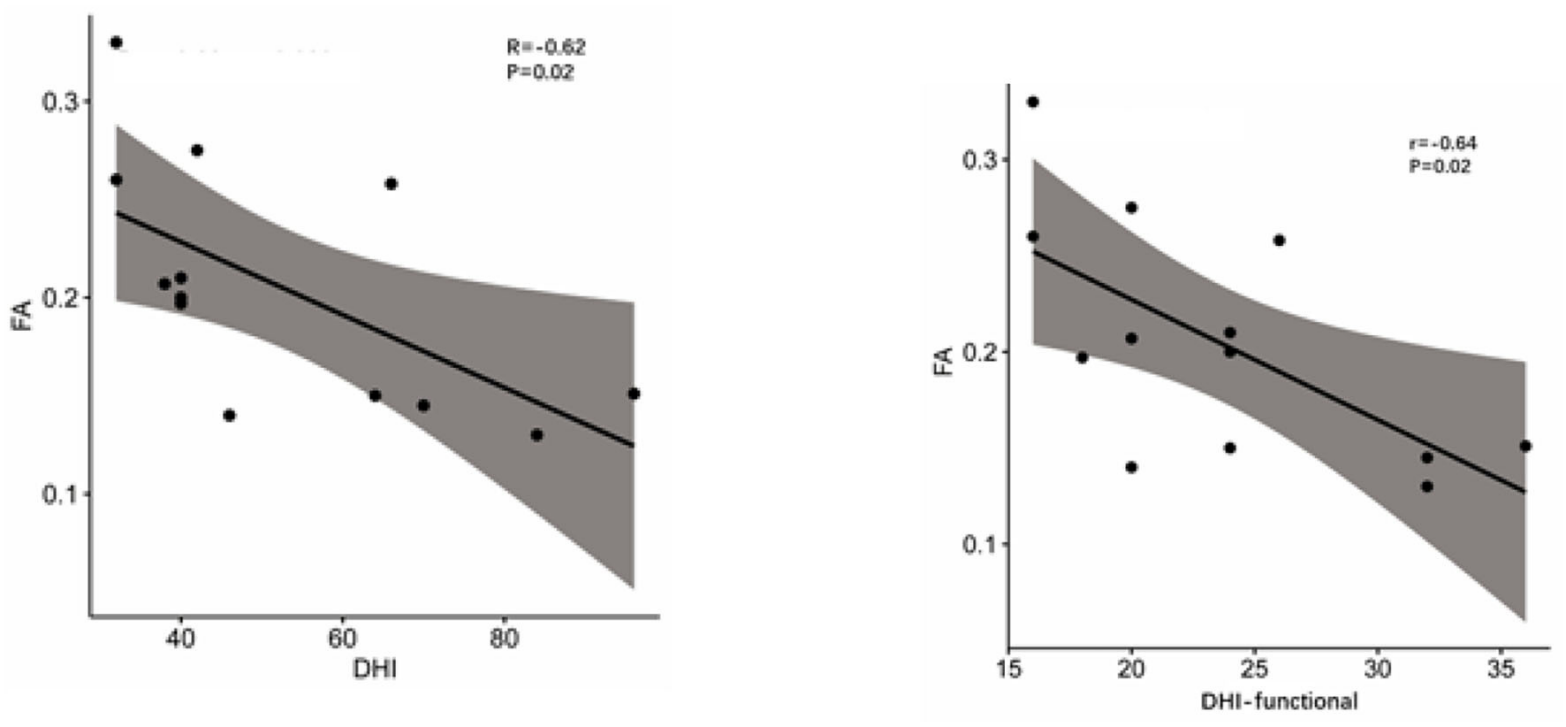
Pearson correlation between FA and DHI and DHI-functional scores.

The DHI score and FA of VCN showed a negative correlation (*r* = −0.62, *P* = 0.02), so as the DHI-functional score and FA value (*r* = −0.64, *P* = 0.02, Figure 6), which indicates the more serious of the dizziness, the lower the FA was detected.

## Discussion

In this work, we have used diffusion tensor to investigate the microstructural properties of the vestibulocochlear nerve. A lower FA and a higher ADC were found in the vestibulocochlear nerve in MD patients compared with healthy controls. The decrease in FA and increase in ADC were presented on both the deafness side and the other side. Further, the FA and DHI had a negative correlation in patients with MD.

FA is highly sensitive to the change in microstructure, decreased FA was related to changes of neural fiber integrity, myelin, and axonal integrity (23, 28, 29). The increase in ADC was shown to be associated with inflammation, edema, and axonal injury (30–32). In summary, the decreased FA and increased ADC may indicate microstructural changes occurred in vestibulocochlear nerves, which may be edema, inflammation, the damage of neural fiber integrity, myelin, or axonal integrity in active MD.

In this research, DTI parameters were analyzed by the affected side or the average value of the two sides in bilateral MD patients. The FA of VCN was lower than that of FN in MD, while they showed no difference in the healthy control group. This suggested that DTI acquisition could provide stable DTI measures in the cisternal segment. FA and ADC showed alterations in VCN may be associated with its special anatomical structure, the cisternal segment of the vestibulocochlear nerve is the transition pathway from the central nerve to the peripheral nerve, and the nerve sheath is thin and easily damaged.

In patients with unilateral deafness, DTI parameters showed no difference on both sides in MD, which indicated that the alteration may originate from both the vestibular nerve fibers and the cochlear nerve fibers. On one hand, recent work on patients with unilateral hearing loss showed FA decrease on both sides, indicating bilateral changes in microstructure of the auditory nerve and auditory pathway in unilaterally deaf subjects (21, 23). On the other hand, the attack of vertigo is always associated with abnormal vestibular signals, whether there were subtle changes in vestibular nerve fibers was unknown. In our research, the decreased FA had negative correlation with DHI and DHI-functional scores. There was no correlation between FA and DHI-emotional, so as FA and DHI-physical. The DHI is very commonly used to qualify the life measure for vestibular disorders and is quite efficient in evaluating the severity of dizziness (33). It means that patients with higher DHI and DHI-functional scores had lower FA, which could indicate a change in vestibular nerve fibers.

## Limitations

The sample size in this study was small, which may affect the result of whether there was a DTI parameter difference between the two sides. However, alterations in FA and ADC were found in vestibulocochlear nerve in patients, suggesting that there were possible changes in the microstructure of the vestibulocochlear nerves in patients with MD. The pathophysiological mechanism of the alterations in DTI was unclear. The separation of the different nerves was processed on T2 map, the counterpoint was processed by the Ziosoft automatically and cloud be manually corrected. Since the nerve cross-section is small, the software cannot observe FA and ADC values with multi-points. To more specifically image the vestibulocochlear nerve, a high-resolution T2WI with 0.5 mm slice thickness, and a DTI technique with 31 directions were used.

## Conclusion

Decreased FA and increased ADC were detected in the vestibulocochlear nerve in patients with MD, and the change in FA was correlated with DHI, these results indicated possible changes in the microstructure of the vestibulocochlear nerve in MD. DTI may be a potential imaging biomarker for the diagnosis of MD.

## Data availability statement

The original contributions presented in the study are included in the article, further inquiries can be directed to the corresponding authors.

## Ethics statement

The studies involving human participants were reviewed and approved by Ethics Committee of Dongzhimen Hospital, Beijing University of Chinese Medicine. The patients/participants provided their written informed consent to participate in this study.

## Author contributions

XY: design and conceptualization of study, acquired data, and drafted the manuscript. ZC: design and conceptualization of study and revised the manuscript for intellectual content. XL: design and conceptualization of study and acquired data. YX: formal analysis and validation. LZ: design and conceptualization of study and supervision. ZY: resources and supervision. All authors contributed to the article and approved the submitted version.

## Funding

This study was supported by Beijing Administration of Traditional Chinese Medicine (BJZYY2019) and Beijing University of Chinese Medicine (2017JYBJS066).

## Conflict of interest

The authors declare that the research was conducted in the absence of any commercial or financial relationships that could be construed as a potential conflict of interest.

## Publisher's note

All claims expressed in this article are solely those of the authors and do not necessarily represent those of their affiliated organizations, or those of the publisher, the editors and the reviewers. Any product that may be evaluated in this article, or claim that may be made by its manufacturer, is not guaranteed or endorsed by the publisher.

## References

[1] Gürkov R Pyykö I Zou J Kentala E. What is Menière's disease? A contemporary re-evaluation of endolymphatic hydrops. J Neurol. (2016) 263(Suppl 1):S71–81. 10.1007/s00415-015-7930-1 27083887 PMC4833790

[2] Hallpike CS Cairns H. Observations on the pathology of Meniere's syndrome. Proc R Soc Med. (1938) 31:1317–36. 19991672 10.1177/003591573803101112 PMC2076781

[3] Niyazov DM Andrews JC Strelioff D Sinha S Lufkin R. Diagnosis of endolymphatic hydrops *in vivo* with magnetic resonance imaging. Otol Neurotol. (2001) 22:813–7. 10.1097/00129492-200111000-00017 11698801

[4] Naganawa S Suzuki K Yamazaki M Sakurai Y Ikeda M. Time course for measuring endolymphatic size in healthy volunteers following intravenous administration of gadoteridol. Magn Reson Med Sci. (2014) 13:73–80. 10.2463/mrms.2013-0080 24769637

[5] Zou J Pyykkö I. Calcium metabolism profile in rat inner ear indicated by MRI after tympanic medial wall administration of manganese chloride. Ann Otol Rhinol Laryngol. (2016) 125:53–62. 10.1177/0003489415597916 26229013

[6] Yamazaki M Naganawa S Tagaya M Kawai H Ikeda M Sone M. Comparison of contrast effect on the cochlear perilymph after intratympanic and intravenous gadolinium injection. AJNR Am J Neuroradiol. (2012) 33:773–8. 10.3174/ajnr.A2821 22173762 PMC8050462

[7] Zhang W Hui L Zhang B Ren L Zhu J Wang F. The correlation between endolymphatic hydrops and clinical features of Meniere disease. Laryngoscope. (2021) 131:E144–50. 10.1002/lary.28576 32083730

[8] Seo YJ Kim J Choi JY Lee WS. Visualization of endolymphatic hydrops and correlation with audio-vestibular functional testing in patients with definite Meniere's disease. Auris Nasus Larynx. (2013) 40:167–72. 10.1016/j.anl.2012.07.009 22867525

[9] Wu Q Dai C Zhao M Sha Y. The correlation between symptoms of definite Meniere's disease and endolymphatic hydrops visualized by magnetic resonance imaging. Laryngoscope. (2016) 126:974–9. 10.1002/lary.25576 26333096

[10] Seo YJ Kim J Kim SH. The change of hippocampal volume and its relevance with inner ear function in Meniere's disease patients. Auris Nasus Larynx. (2016) 43:620–5. 10.1016/j.anl.2016.01.006 26856304

[11] Basser PJ Mattiello J LeBihan D. MR diffusion tensor spectroscopy and imaging. Biophys J. (1994) 66:259–67. 8130344 10.1016/S0006-3495(94)80775-1 PMC1275686

[12] Stejskal E Tanner J. Spin diffusion measurements: spin echoes in the presence of a time-dependent field gradient. J Chem Phys. (1965) 42:288e292

[13] Moseley ME Cohen Y Kucharczyk J Mintorovitch J Asgari HS Wendland MF. Diffusion-weighted MR imaging of anisotropic water diffusion in cat central nervous system. Radiology. (1990) 176:439–45. 2367658 10.1148/radiology.176.2.2367658

[14] Beaulieu C. The basis of anisotropic water diffusion in the nervous system: a technical review. NMR Biomed. (2002) 15:435–55. 10.1002/nbm.782 12489094

[15] Basser PJ. Inferring microstructural features and the physiological state of tissues from diffusion-weighted images. NMR Biomed. (1995) 8:333–44. 8739270 10.1002/nbm.1940080707

[16] Pierpaoli C Jezzard P Basser PJ Barnett A Di Chiro G. Diffusion tensor MR imaging of the human brain. Radiology. (1996) 201:637–48. 8939209 10.1148/radiology.201.3.8939209

[17] Puig J Blasco G Schlaug G Stinear CM. Daunis-I-Estadella P Biarnes C. Diffusion tensor imaging as a prognostic biomarker for motor recovery and rehabilitation after stroke. Neuroradiology. (2017) 59:343–51. 10.1007/s00234-017-1816-0 28293701

[18] Kalra S Müller HP Ishaque A Zinman L Korngut L Genge A. A prospective harmonized multicenter DTI study of cerebral white matter degeneration in ALS. Neurology. (2020) 95:e943–52. 10.1212/WNL.0000000000010235 32646955 PMC7668555

[19] Goldberg-Zimring D Mewes AU Maddah M Warfield SK. Diffusion tensor magnetic resonance imaging in multiple sclerosis. J Neuroimag. (2005) 15:68S−81S. 10.1177/1051228405283363 16385020

[20] Otte WM van Eijsden P Sander JW Duncan JS Dijkhuizen RM Braun KP. meta-analysis of white matter changes in temporal lobe epilepsy as studied with diffusion tensor imaging. Epilepsia. (2012) 53:659–67. 10.1111/j.1528-1167.2012.03426.x 22379949

[21] Vos SB Haakma W Versnel H Froeling M Speleman L Dik P. Diffusion tensor imaging of the vestibulocochlear nerve in patients with long-term single-sided deafness. Hear Res. (2015) 323:1–8. 10.1016/j.heares.2015.01.010 25655832

[22] Roundy N Delashaw JB Cetas JS. Preoperative identification of the facial nerve in patients with large cerebellopontine angle tumors using high-density diffusion tensor imaging. J Neurosurg. (2012) 116:697–702. 10.3171/2011.12.JNS111404 22283188

[23] Kim S Kwon HJ Kang EJ Kim DW. Diffusion-tensor tractography of the auditory neural pathway: clinical usefulness in patients with unilateral sensorineural hearing loss. Clin Neuroradiol. (2020) 30:115–22. 10.1007/s00062-018-0733-x 30374668

[24] De Groot M Verhaaren BF de Boer R Klein S Hofman A van der Lugt A. Changes in normal-appearing white matter precede development of white matter lesions. Stroke. (2013) 44:1037–42. 10.1161/STROKEAHA.112.680223 23429507

[25] Kronlage M Pitarokoili K Schwarz D Godel T Heiland S Yoon MS. Diffusion tensor imaging in chronic inflammatory demyelinating polyneuropathy: diagnostic accuracy and correlation with electrophysiology. Invest Radiol. (2017) 52:701–7. 10.1097/RLI.0000000000000394 28574858

[26] Barr K. Electrodiagnosis of lumbar radiculopathy. Phys Med Rehabil Clin N Am. (2013) 24:79–91. 10.1016/j.pmr.2012.08.011 23177032

[27] Jacobson GP Newman CW. The development of the dizziness handicap inventory. Arch Otolaryngol Head Neck Surg. (1990) 116:424–7. 2317323 10.1001/archotol.1990.01870040046011

[28] Heckel A Weiler M Xia A Ruetters M Pham M Bendszus M. Peripheral nerve diffusion tensor imaging: assessment of axon and myelin sheath integrity. PLoS ONE. (2015) 10:e0130833. 10.1371/journal.pone.0130833 26114630 PMC4482724

[29] Morisaki S Kawai Y Umeda M Nishi M Oda R Fujiwara H. *In vivo* assessment of peripheral nerve regeneration by diffusion tensor imaging. J Magn Reson Imag. (2011) 33:535–42. 10.1002/jmri.22442 21287654

[30] Werring DJ Clark CA Barker GJ Thompson AJ Miller DH. Diffusion tensor imaging of lesions and normal-appearing white matter in multiple sclerosis. Neurology. (1999) 52:1626–32. 10331689 10.1212/wnl.52.8.1626

[31] Field AS Alexander AL. Diffusion tensor imaging in cerebral tumor diagnosis and therapy. Top Magn Reson Imag. (2004) 15:315–24. 10.1097/00002142-200410000-00004 15627005

[32] Lin TH Spees WM Chiang CW Trinkaus K Cross AH Song SK. Diffusion fMRI detects white-matter dysfunction in mice with acute optic neuritis. Neurobiol Dis. (2014) 67:1–8. 10.1016/j.nbd.2014.02.007 24632420 PMC4035476

[33] Van De Wyngaerde KM Lee MK Jacobson GP Pasupathy K Romero-Brufau S McCaslin DL. The component structure of the dizziness handicap inventory (DHI): a reappraisal. Otol Neurotol. (2019) 40:1217–23. 10.1097/MAO.0000000000002365 31469793

